# Fatigue Crack Length Estimation Using Acoustic Emissions Technique-Based Convolutional Neural Networks

**DOI:** 10.3390/s26020650

**Published:** 2026-01-18

**Authors:** Asaad Migot, Ahmed Saaudi, Roshan Joseph, Victor Giurgiutiu

**Affiliations:** 1 Department of Petroleum and Gas Engineering, College of Engineering, University of Thi-Qar, Nasiriyah 64001, Iraq; 2 Department of Artificial Intelligence Engineering, College of Artificial Intelligence and Cyber Security Engineering, University of AL-Muthanna, Samawah 66001, Iraq; 3 Department of Mechanical Engineering, University of South Carolina, 300 Main Street, Columbia, SC 29208, USA; 4 Department of Mechanical Engineering, University of Alabama, Tuscaloosa, AL 35906, USA

**Keywords:** fatigue crack growth, CNN, acoustic emission, SHM, deep learning, crack length estimation

## Abstract

Fatigue crack propagation is a critical failure mechanism in engineering structures, requiring meticulous monitoring for timely maintenance. This research introduces a deep learning framework for estimating fatigue fracture length in metallic plates through acoustic emission (AE) signals. AE waveforms recorded during crack growth are transformed into time-frequency images using the Choi–Williams distribution. First, a clustering system is developed to analyze the distribution of the AE image-based dataset. This system employs a CNN-based model to extract features from the input images. The AE dataset is then divided into three categories according to fatigue lengths using the K-means algorithm. Principal Component Analysis (PCA) is used to reduce the feature vectors to two dimensions for display. The results show how close together the data points are in the clusters. Second, convolutional neural network (CNN) models are trained using the AE dataset to categorize fracture lengths into three separate ranges. Using the pre-trained models ResNet50V2 and VGG16, we compare the performance of a bespoke CNN using transfer learning. It is clear from the data that transfer learning models outperform the custom CNN by a wide margin, with an accuracy of approximately 99% compared to 93%. This research confirms that convolutional neural networks (CNNs), particularly when trained with transfer learning, are highly successful at understanding AE data for data-driven structural health monitoring.

## 1. Introduction

The growing prevalence of aging engineering structures and diverse operational environments necessitates the development of a scientifically robust and effective health monitoring technology. Fatigue crack growth in aeronautical, civil, and mechanical structures is of critical importance since the undetected existence of cracks in such structures can result in a catastrophic failure. Early finding and precise prediction of crack size are critical for the reliability of the components and avoiding surprise failure. Conventional non-destructive testing (NDT) techniques, including ultrasonic testing, eddy current inspection, and X-ray visualization, are proficient in the measurement tasks, but the intervention of the manual operation prevents them from real-time monitoring applications [1,2].

As a passive method of structural health monitoring (SHM), acoustic emission (AE) is a growing technique that is being used to monitor damages like fatigue cracking. The AE method has gained recognition among structural health monitoring and non-destructive evaluation (NDE) professionals, and it has been continuously improved since it was first developed [3,4,5]. Fatigue crack growth is an issue frequently encountered in metal structures. The issue of fatigue-crack growth becomes worse as the metallic structure gets older. When fatigue-crack growth occurs in metallic structures, the formation of cracks releases AE signals. Different researchers have studied the properties of AE signals as fatigue-crack growth progresses with the help of different sensors. Very few studies have been conducted on the relationship between the crack length and the AE signal signatures. Accurate measurement of crack length is critical in the scheduling of maintenance of the structure where the crack is propagating [6,7,8]. The aim of this research was to develop a new technique based on Convolutional Neural Networks (CNN) to quantify AE fatigue crack length by analyzing AE signals captured during the fatigue crack growth process with a single PWAS.

The phenomenon of AE has been used for the damage assessment of structures for the detection and location of the sources of fatigue cracks [9,10]. The AE method is considered a passive and wave propagation-based SHM method of monitoring a structure in situ. The initiation and growth of fatigue cracks as a result of cyclic loading is a common form of damage in metallic structures. The cyclic loading will generate AE waveforms that can be studied. There has been a lot of interest in the study of the acoustic emissions from the fatigue crack growth for the last few years [3,11]. Quy and Kim [12] present a new method for detecting and localizing crack in high-pressure fluid pipelines using received acoustic emission signals from two sensors. In this work, the localization accuracy can be improved by canceling false sources based on energy attenuation. The presented result shows the ability of this method for identifying fault locations and structural changes. Rizzo and Enshaeian [13] achieve an extensive look at the main problems of bridge structural health monitoring (SHM), analyzing the systems that have been utilized on USA bridges over the past two decades. It investigates how different sensing technologies and data-analysis methods are used to detect key degradation mechanisms, including stiffness loss, time- and temperature-dependent effects, fatigue, corrosion, scour, and impact events. The authors emphasize the strong influence of environmental effects on measured responses and the need for robust data interpretation supported by finite element modeling. Pascoe et al. [14] studied the phenomena associated with the behavior of fatigue crack growth events in the context of a single fatigue cycle. This investigation provided insight into the fact that crack growth may take place during both loading and unloading phases, provided the release rate of strain energy surpasses a critical threshold level. Bhuiyan and colleagues [15] explored the AE signal patterns captured by installed sensors during experiments on fatigue crack growth in thin metallic plates. In their study, they recorded AE signals at a slow fatigue loading frequency of less than 0.25 Hz, focusing on a brief period of crack length advancement. They identified eight distinct signal signatures that were associated with the processes of crack growth. Roberts and Talebzadeh [16] explored the relationship between the rates of acoustic emissions and the rates at which cracks propagate. Researchers have employed a range of signal processing methods and clustering techniques to distinguish between AE signals that arise from different activities [17]. They have also explored the relationships between crack characteristics and the features of AE signals using various analytical approaches [18,19]. There are a variety of techniques that have been created to identify damage in engineering structures by analyzing how waves interact with that damage [20,21]. The Piezoelectric wafer active sensor, often referred to as PWAS, is commonly used to sense AE signals [22,23]. This transducer is recognized for its great characteristics, including high sensitivity and reliable stability. Researchers have successfully utilized clay boundaries to minimize wave reflections from plate boundaries in ultrasonic experiments. These clay boundaries have proven effective in reducing reflections from AE signals during low cycle-fatigue tests [15].

Although there has been much reported about sensors for the capturing of AE waves, and even analysis in terms of wave characteristics, not much work has been done to relate them to the length of a fatigue crack and AE waveform signatures. However, some intriguing initial work by Joseph et al. [6] offered both analytical and experimental evidence suggesting that there might be a connection between fatigue crack AE signals and crack length. The basic idea is that when energy is released from the fatigue crack, it resonates with the crack itself, creating a standing wave pattern. Garrett et al. [8] have developed an innovative AI-driven signal analysis system that can identify AE wave information and predict the length of the crack from which it originates. With this new capability, monitored engineered structures can undergo on-demand maintenance instead of routine maintenance, which might have a significant impact on many different businesses.

Recent investigations have focused on the capabilities of artificial intelligence, which have been applied in comparable manners. Clustering techniques in machine learning were employed to examine different fracture mechanisms in fatigue-loaded wind turbine blades. Furthermore, these techniques facilitated the correlation of damage modes in adhesive composite joints subjected to tensile acoustic emission tests [24,25]. Li et al. [26] presented a new method of structural damage identification based on strain. This study uses both inverse finite element method (iFEM) and strain mode differences based on a convolutional neural network to detect structural defects. The results demonstrate that the method is efficient, robust to noise, and well-suited for practical structural health monitoring applications. Ai et al. [27] achieved an automated acoustic emission–based damage localization method that estimates damage coordinates using only a single sensor, reducing cost and deployment constrain. It studies the challenge of long-term monitoring of stainless steel canisters used to store spent nuclear fuel, which are susceptible to stress corrosion cracking, especially in coastal environments. By fusing waveform, frequency-domain, entropy, and time–frequency features into image representations and analyzing them with a weighted ensemble CNN regression model, the method achieves accurate damage localization. Noor and David [23] achieve a deep review of utilizing the acoustic emission (AE) technique for structural health monitoring (SHM) of composite structures. It delineates the essential concepts of AE, critical signal attributes, damage mechanisms in composites, and the identification of various failure modes through AE characteristics. The paper shows significant advantages of AE for real-time damage detection in composite structures while addressing present issues relating to anisotropy, noise, and signal interpretation. Migot et al. [28] employed a specially designed CNN model with scanning laser Doppler vibrometer (SLDV) experiments on composite plates to predict the delamination depth with remarkable accuracy. Ramasso et al. [29] employed signal clustering along with pattern recognition machine learning algorithms to categorize AE signals into specific groups. A new time-aware clustering validity index (CVI) was presented for SHM. It utilizes Kullback–Leibler divergence in conjunction with acoustic emission data to detect preliminary indicators of damage [30]. The methodology has been corroborated by experiments on bolt loosening, micro-drilling, and hybrid joints. This approach surpasses conventional CVIs in both precision and practical application for failure prevention. Tao and colleagues [31] developed an automated model for characterizing fatigue damage in composite structures using deep learning convolutional neural networks. Nasiri and Khosravani [32] utilized machine learning techniques to forecast how additively manufactured components would perform structurally and how they might fracture. Wiangkham et al. [33] explore the use of artificial neural networks (ANN) and adaptive neuro-fuzzy inference systems (ANFIS) to predict fracture toughness across polymethyl methacrylate material (PMMA), taking into account specimen dimensions and loading angles. The AI models provide accurate estimates of fracture toughness, with their predictions aligning closely with experimental results. This highlights the potential of AI for dependable material property predictions in testing scenarios.

Combination SHM paradigm, making it clear that artificial intelligence techniques based on AE are better seen as additions to vibration-based damage assessment than as replacements. This kind of integration is a natural and crucial step for future study and practical use of SHM. Huang et al. [34] present a framework for structural damage identification that combines modal frequency strain energy assurance criteria, modal flexibility, and an improved moth–flame optimization algorithm to overcome the inefficiencies and limited sensitivity of conventional dynamic-based approaches. Benchmark testing and numerical case studies under noise and temperature fluctuations indicate that the improved optimization surpasses other metaheuristic methods and yields superior damage identification accuracy compared to traditional indicators. Experimental validations on beam and frame structures further substantiate the method’s resilience to ambient noise and its efficacy in accurately identifying symmetric structural damage. A two-stage methodology was developed [35] for detecting degradation in bridge rubber bearings to rectify the limitations of traditional detection techniques and the lack of research on bearing health. The method first introduces a novel relative modal strain energy indicator to accurately detect bearing damage and then combines this information with sailfish optimization to determine how bad the damage is. Numerical, experimental, and real-bridge case studies demonstrate that the method is robust against noise and effective for both simple and complex bridge designs, offering a practical and economical solution for assessing bearing damage.

Planning maintenance for structures exhibiting indications of crack propagation requires obtaining an accurate assessment of the crack’s length. The objective of this work was to develop an AI system capable of estimating the crack length based on AE waveforms. The differences between the current work and the presented methods (Garrett et al. [8] and Ennis et al. [36]) can be boiled down to three main points:

1. Propose a CNN-K Means-PCA clustering system to analyze the non-linear characteristics of AE signals.
2. A large database of acoustic emission (AE) signals that include AE signatures directly correlated with fatigue crack length values was utilized.
3. Using a proposed two-dimensional convolutional neural network (CNN) and transfer learning models to estimate crack lengths based on acoustic emission (AE) signal signatures.

## 2. Test Specimen Preparation and Experimental Setup

**Figure 1:**
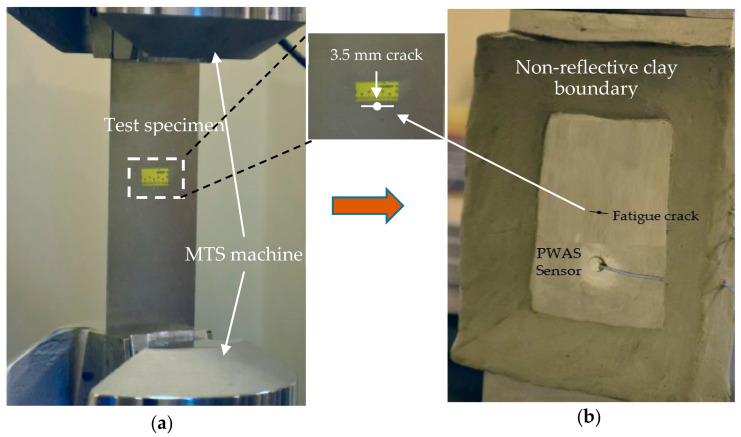
The preparation of the test plate: (**a**) a 3.5 mm tip-to-tip crack length was created at 322 kcycles of fatigue loading using the MTS machine; (**b**) an AE test specimen was bonded with the PWAS. Non-reflective clay boundaries (NRB) were provided on the specimen to avoid the reflection of AE signals from the specimen boundaries.

In this study, the design of an experimental specimen to capture acoustic emissions (AE) during crack growth in a thin metallic plate was conducted. We chose aluminum 2024-T3, a popular material in aircraft manufacturing, to prepare the test specimen. From a larger plate of this aluminum, we machined coupons measuring 103 mm in width, 305 mm in length, and 1 mm in thickness using a shear metal cutting machine. The specimen is wide enough to allow a long crack to develop in the center, where a 1 mm hole was drilled. The test specimen was mounted on an MTS machine to apply fatigue cyclic loading, with loads ranging from 1.38 to 13.85 kN at a frequency of 10 Hz. This continuous loading caused a fatigue crack to originate from the hole. After 322,000 cycles, the crack length measured was 3.5 mm (as shown in Figure 1a). The experiment was stopped, and the specimen was removed from the MTS machine. After that, a piezoelectric wafer active sensor (PWAS) was installed, and a non-reflective clay boundary (NRB) was provided to the specimen (see Figure 1b). The PWAS was strategically placed at a distance of 25 mm from the crack. The NRB effectively reduced the reflections of the AE signals originating from the plate’s edges, enabling the acquisition of clear, reflection-free signals. The specimen’s broad geometry was deliberately designed to facilitate the propagation of acoustic waves over an extended distance to the edges. The selected design resulted in the signals dissipating after reflecting off the boundaries, prior to reaching the sensor, as a consequence of geometric spreading and material damping.

**Figure 2:**
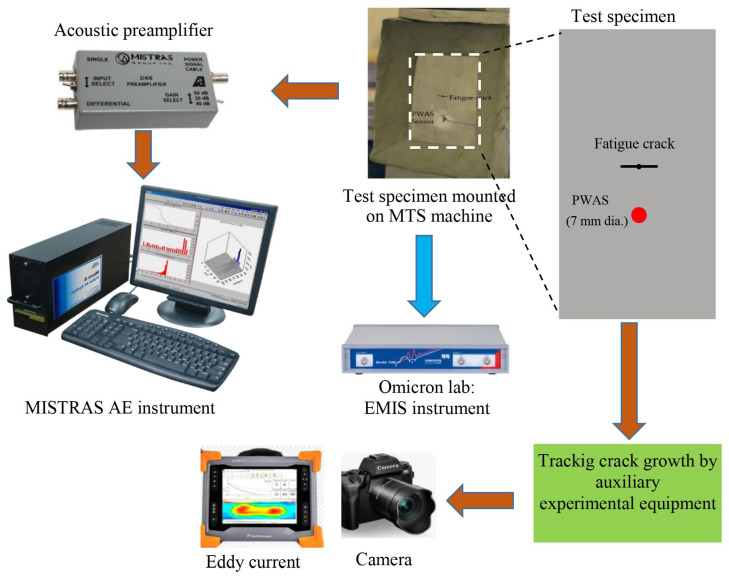
The experimental setup of recording acoustic emission (AE) data during a fatigue-crack occurrence. This configuration is intended to efficiently monitor and analyze the signals generated during the crack-forming process.

Subsequent to the installation of the PWAS transducer and the establishment of the non-reflective clay border (NRB), the test specimen was reinserted into the MTS machine for additional fatigue loading while monitoring the AE waveforms. Figure 2 illustrates the experimental configuration employed to capture the acoustic emission signals from a fatigue crack development event. To verify the bond quality of the PWAS transducer, we routinely conducted assessments utilizing electromechanical impedance spectroscopy (EMIS) [6]. The PWAS transducer detected acoustic emission signals during crack propagation events and was linked to an acoustic preamplifier. The sensor was connected to the acoustic preamplifier. This preamplifier functions as a bandpass filter, selectively processing signals within the range of 30 kHz to 700 kHz. With selectable gains of 20/40/60 dB via a switch, it can work with either a single-ended or differential sensor. For this experiment, a 40 dB gain was opted for. The preamplifier was linked to the MISTRAS MICRO-II AE system with a sampling frequency of 10 MHz to capture any high-frequency AE signals. The MISTRAS MICRO-II system used has the capability of holding up to 32 AE channels. The system used four Physical Acoustics’ PCI-8 acoustic emission systems on a board. The PCI-bus provided AE data transfer rates up to 132 Mb/sec to the PC. The timing parameters for the MISTRAS system were a peak definition time of 200 µs, a hit definition time of 800 µs, and a hit lockout time of 1000 µs. We continued to grow the crack by an additional 5.9 mm, bringing the total crack length to 9.5 mm from tip to tip, all while diligently recording the AE signals. As illustrates in Figure 2, the experimental auxiliary apparatus, including the camera and eddy current devices, was employed for the continuous monitoring of crack propagation. The camera consistently recorded video of the crack tips alongside measurement tape affixed to the test material, while an eddy current probe from Eddyfi Technologies^®^ (https://www.eddyfi.com/) was intermittently employed to measure the crack length.

## 3. Experimental Result and Discussion

Following the installation of the acoustic emission (AE) equipment on the specimen, fatigue loading began with a minimum load of 1.38 kN, a maximum load of 13.85 kN, and a frequency of 2 Hz. In this experiment, an additional 188,000 cycles of fatigue loading were applied, resulting in a total crack length of 9.5 mm from tip to tip. The AE system (see Figure 2) actively recorded emission events throughout the loading process. The initiation, acquisition, and processing of crack growth acoustic emission signals can be summarized in five steps:

1. Micro-crack growth and wave emission—strain waves travel from the tips of cracks as micro-cracks develop.
2. Signal detection—The PWAS transducer picks up the event, while the AEWin™ software (https://www.physicalacoustics.com/by-product/aewin/ (accessed on 20 December 2025)) records the signal.
3. Signal storage and preprocessing—The raw time-domain waveform is saved and ready for analysis. A noise cancelation algorithm helps filter out any system-generated noise.
4. Frequency-domain analysis—The filtered signal goes through a Fast Fourier Transform (FFT) to pull out frequency-domain characteristics.
5. Time-frequency analysis—The Choi–Williams transform (CWT) was applied to the filtered signals. The Choi–Williams distribution (CWD) is a member of the Cohen class. It is connected to things like the instantaneous median frequency and the instantaneous power, which is the sum of all frequencies at each time [37]. This CWT is a form of wavelet transform that produces an intensity plot, providing simultaneous information regarding both the time and frequency domains of the acoustic emission wave.

The continuous acoustic emission monitoring system detects the release of an acoustic emission from the crack, as illustrated in Figure 2. As the crack lengthens, the system identifies an increasing number of acoustic emission events. The occurrence of this phenomenon is attributed to the frictional conditions established by the crack surfaces, which are recognized as the primary cause of acoustic emissions in scenarios involving fatigue cracks [36].

**Figure 3:**
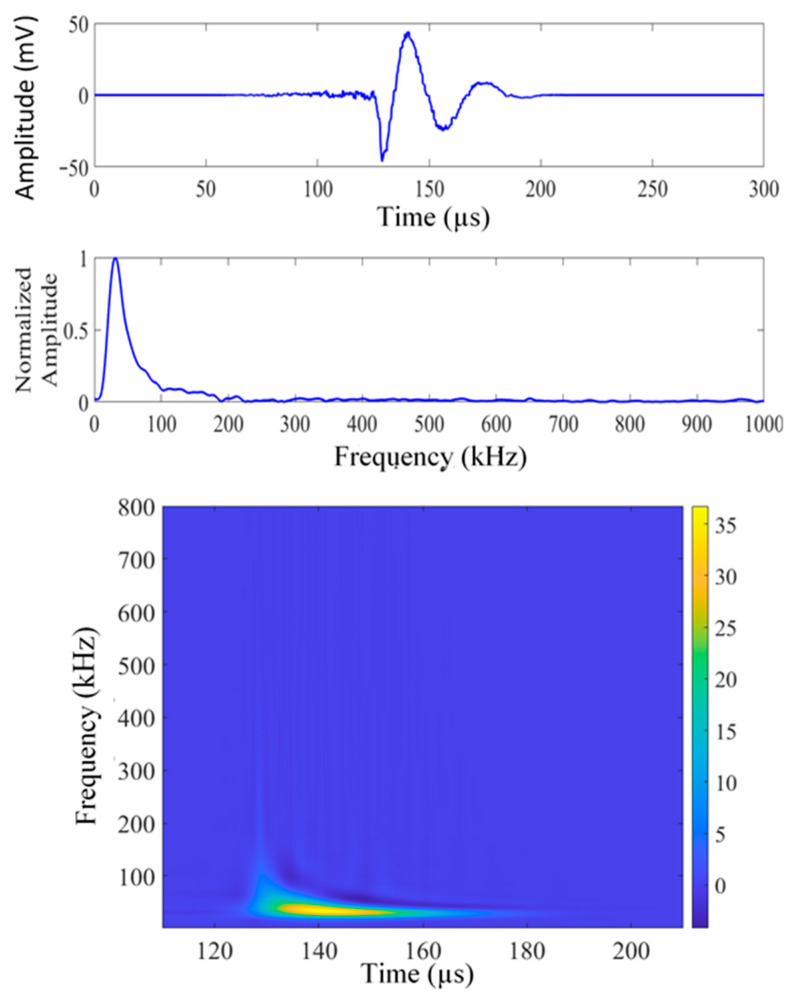
Time domain, frequency domain, and Choi–Williams transform (CWT) of experimental signal #12, 4–8 kcycles (crack length about 4 mm).

**Figure 4:**
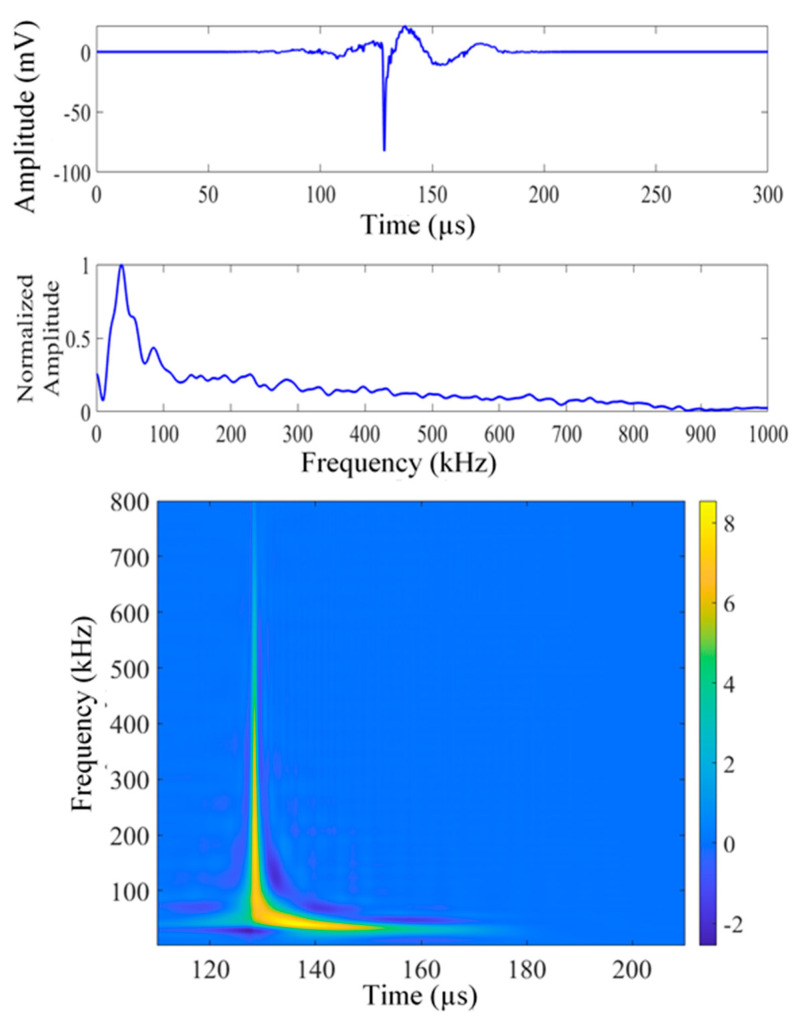
Time domain, frequency domain, and Choi–Williams transform (CWT) of experimental signal #73, 28–32 kcycles (crack length about 6 mm).

**Figure 5:**
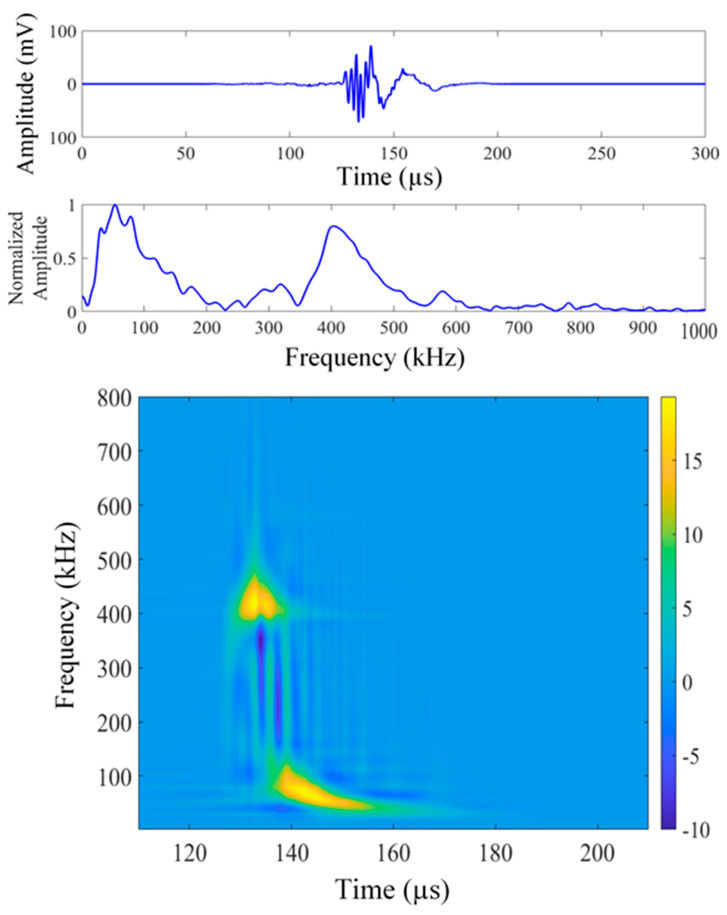
Time domain, frequency domain, and Choi–Williams transform (CWT) of experimental signal #52, 64–68 kcycles (crack length about 8 mm).

**Figure 6:**
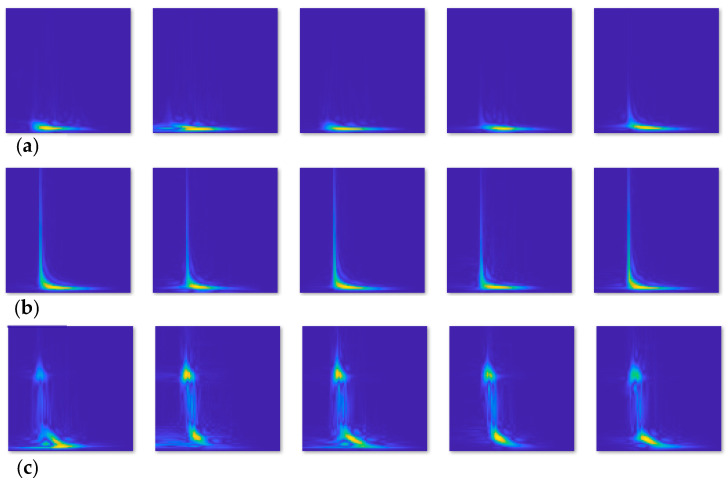
Experimental Choi–Williams transform (CWT) images (224 × 224 pixels) of AE signals were used as input datasets to the proposed CNN models. These images are classified based on the crack length into three classes: (**a**) class A (208 images); (**b**) class B (560 images); (**c**) class C (520 images).

Figure 3, Figure 4 and Figure 5 present a sample set of experimental acoustic emission signals corresponding to crack lengths of 4, 6, and 8 mm. The noise in the signal was numerically removed by evaluating the frequency spectrum of the noise prior to the signal and canceling the noise from the signal in the frequency domain. The Choi–Williams transform (CWT) was employed to analyze the AE signals within the time-frequency domain. The sigma parameter of CWD was chosen as 30 for the analysis, and a sampling frequency of 10 MHz was used for plotting the CWT. The CWT effectively consolidates time and frequency information into an intensity plot. This method is particularly useful in the development of artificial intelligence tools for signal analysis. Figure 3 illustrates the AE signal corresponding to a crack length of 4 mm (AE hit #12 from the fatigue cycle range of 4–8 kcycles). The displayed signal encompasses the complete collection of signals obtained at a crack length of 3.5–5.5 mm, all exhibiting analogous frequency peaks. This signal consistently exhibits a common frequency peak at approximately 30 kHz. Refer to Figure 4, which illustrates the AE signal corresponding to a crack length of 6 mm (AE hit #73 from the fatigue cycle range of 28–32 kcycles). The displayed signal represents the comprehensive collection of signals obtained within the crack length range of 5.5–7.5 mm. This signal clearly indicates a consistent frequency peak near 36 kHz, accompanied by a cluster of minor peaks. Refer to Figure 6, which depicts the AE signal corresponding to a crack length of 8 mm (AE hit #52 from the fatigue cycle range of 64–68 kcycles). This signal denotes the comprehensive dataset gathered within the crack length range of 7.5 to 9.5 mm. These signals consistently exhibit two prominent frequency peaks: one at 52 kHz and another at 403 kHz. Continuous fatigue loading may cause the disbonding of the PWAS from the specimen, potentially leading to the detection of misleading AE signals. We assessed the bonding quality of the PWAS to the specimen through electromechanical impedance spectroscopy (EMIS) during the fatigue loading process.

The CWT output is essential as it connects raw AE data with convolutional neural network (CNN) processing, facilitating AI-driven analysis of acoustic emission signals.

## 4. Datasets for CNN Models

When it comes to Structural Health Monitoring (SHM), data is absolutely essential. With the rise in deep learning techniques that can process and analyze immense amounts of data, it is become increasingly clear just how crucial reliable data is for effective SHM systems [38]. To create the CNN system focused on predicting crack length, we utilized the AE signals gathered from the experiment outlined in Section 3.

In this work, three different datasets consisting of images were prepared from experimental AE signals captured during crack growth when the crack was in the ranges of 3.5–5.5 mm, 5.5–7.5 mm, and 7.5–9.5 mm in total length, as presented in Table 1. A MATLAB R2016a code was performed to process these time-domain signals to generate time-frequency plots using the Choi–Williams transform (CWT). The second MATLAB code was prepared to customize and increase the resolution of the images, crop and resize the images to be 224 × 224 pixels before being used as input data to the designed CNN models. The dataset included a total of 1288 images (CWT figures). Out of these, 208 were derived from signals of crack lengths ranging from 3.5 mm to 5.5 mm (class A), 560 were derived from signals of crack lengths ranging from 5.5 mm to 7.5 mm (class B) while the remaining 520 were from signals with crack lengths between 7.5 mm and 9.5 mm (class C), as illustrated in Figure 6.

## 5. Exploratory Data Analysis Using Clustering

Before using supervised learning, it is very important to cluster acoustic emission (AE) data to understand the patterns and structures of the data samples. Clustering facilitates the identification of potential stages of fatigue fracture development by grouping similar signal patterns based on their inherent properties, eliminating the need for labeled data. [39]. This is especially helpful when getting crack length labels is too expensive, takes too long, or is impossible. Unsupervised clustering can help us understand how AE signals naturally separate from each other. This information can then be used to create classification algorithms and feature extraction models. Clustering can also be used to explore data and find outlier signals or infrequent failure modes that would not be seen otherwise. For example, signals that are gathered together in a different cluster may be related to distinctive acoustic occurrences like micro-cracks or rapid changes in how cracks spread. This not only helps with organizing datasets, but it also helps with training models by showing scenarios that might need to be treated differently or modeled separately. So, clustering connects raw AE data to deep learning models, which helps in understanding how the data is spread out and what its underlying structures are. The next few parts show how the K-means algorithm works and how it may be utilized with a deep learning model and Principal Component Analysis (PCA).

### 5.1. K-Means Clustering Methodology

The K-Means is a popular unsupervised clustering algorithm that divides data into k different groups based on how similar the features are. When it comes to acoustic emission (AE) data, each AE signal is shown as a vector in a high-dimensional feature space (for example, taken from time-frequency representations), and k-means tries to locate natural groups of these vectors. The technique starts by randomly choosing *k* centroids and then allocating each data point to the closest centroid. It then updates the centroids to be the mean of the points that were allocated to them. This procedure is reiterated until convergence is achieved. The goal of K-Means is to lower the within-cluster sum of squares (WCSS), which is also called the distortion cost [40,41]:

(1) $$J=\sum_{i=1}^{k}\sum_{x_{j}\in c_{i}}{\left\|x_{j}-\mu_{i}\right\|}^{2}$$

where $C_{i}$ is the *i-*th cluster, $\mu_{i}$ is the centroid of cluster *i*, and $x_{j}$ is a point in that cluster.

### 5.2. Elbow Method to Choose the Number of Centroids

**Figure 7:**
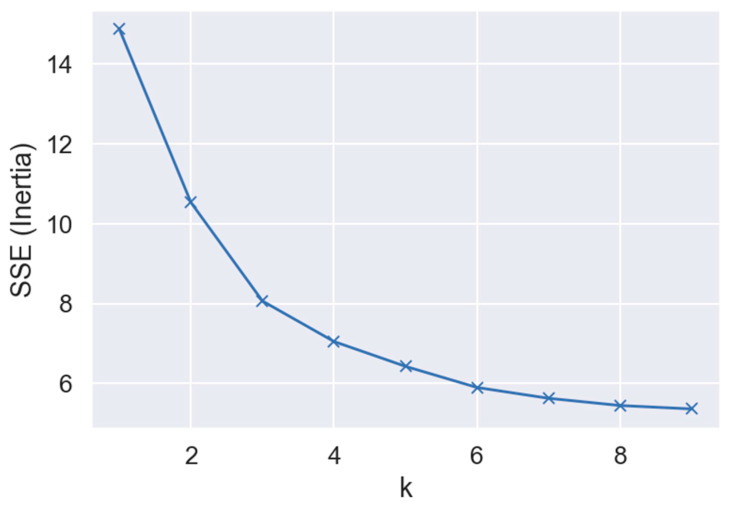
The curve of the Elbow method on the AE dataset. In this plot, *k* represents the number of clusters, and the SSE shows the sum of squared errors for each *k* value.

Determining the ideal number of clusters, *k*, is essential for the efficacy of the K-Means algorithm. A prevalent technique is the elbow method, which entails calculating the total WCSS for various *k* values and graphing these results to illustrate the balance between model complexity and fit. As *k* increases, WCSS typically decreases because more clusters result in each being smaller and more specific. However, after a certain point, the rate of decrease sharply levels off—forming an “elbow” in the plot. The value of *k* at this elbow point is considered optimal. The elbow curve with AE data, Figure 7, helps in discovering natural groupings that might correspond to physical stages of crack propagation (e.g., initiation, stable growth, unstable growth). The elbow angle starts bending when the sum of squared error approaches 10 or 8. The value of SSE is decreasing as the number of clusters increases. We know in advance that the AE dataset has three classes (A, B, and C). According to the elbow curve, the SSE value for *k* equals 3, which is 8. However, SSE continues to decrease as *k* increases. This means some of the different-class samples fall within the same cluster. The shortness of the fatigue crack may be the cause of this similarity.

### 5.3. Dimensionality Reduction Using PCA

Principal Component Analysis (PCA) is a widely used linear dimensionality reduction technique that transforms high-dimensional data into a lower-dimensional space while retaining most of its variance [42]. In the domain of acoustic emission (AE) signals, particularly following their transformation into high-dimensional time-frequency representations, PCA functions to streamline the data structure while preserving essential information. This is particularly useful when visualizing clusters or feeding the data into machine learning models that perform better with fewer input features. PCA achieves this by identifying the directions (principal components) in which the data varies the most and projecting the data onto a new coordinate system aligned with those directions.

Mathematically, PCA begins by standardizing the data to have a zero mean and unit variance, then computing the covariance matrix of the features. The eigenvalues and eigenvectors of this matrix are then calculated to identify the principal components. These components are sorted in descending order of their associated eigenvalues, and the top k components are selected to form the new feature space. The transformation of an original feature vector *x* to the PCA-reduced form *x*′ is given by [43,44]:

(2) $$x^{\prime }=W^{T}(x-\mu )$$

where *W^T^* is the transpose matrix of selected eigenvectors (principal components (PCs)), and *μ* is the mean of the dataset.

**Figure 8:**
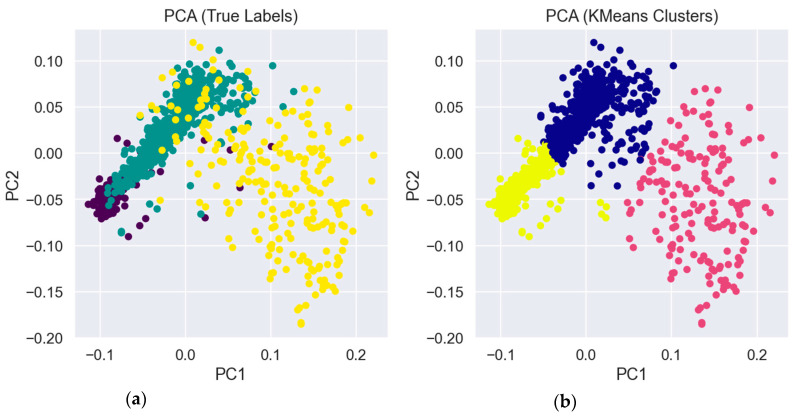
The 2D principal components are visualized using their true labels (**a**), then after applying the K-means algorithm (**b**).

In this study, PCA was used after feature extraction via CNNs or time-frequency transformations to reduce the dimensionality of AE data before applying clustering or visualization techniques such as scatter plots. For instance, a 512-dimensional CNN feature vector was reduced to two principal components for 2D visualization. This facilitated the validation of the effectiveness of clustering techniques such as k-Means and the identification of inherent groupings within the data. Furthermore, PCA functioned as a noise filter by eliminating less informative dimensions, thus augmenting the distinction between crack growth stages and improving the efficacy and interpretability of future classification models. Employing K-Means with *k* = 3 generates three distinct clusters. Nonetheless, numerous samples are misallocated due to some overlaps among these groups, as illustrated in Figure 8.

### 5.4. Cluster Interpretation and Initial Observations

Upon implementing K-means clustering and analyzing the resultant groups through PCA-reduced data, preliminary patterns began to develop within the AE dataset. The clustering results indicated the existence of natural groupings aligned with three classes of fatigue crack lengths. One cluster frequently exhibited signals characterized by diminished amplitude and frequency content, possibly indicative of nascent micro-cracking. Signals from a different cluster showed more frequent and stronger acoustic bursts, which probably indicated more sophisticated crack propagation. These findings supported the usefulness of clustering as a diagnostic technique by offering an unsupervised viewpoint that was in close agreement with the physical predictions of fracture mechanics. In numerous studies, the PCA visualization demonstrated that specific clusters were distinctly separable, producing compact and identifiable groups in the reduced feature space. This distinction signifies that the time-frequency representations obtained from the CNN model effectively encapsulate distinguishable information pertinent to crack length or damage severity. Nevertheless, some intersection across clusters was seen, especially during transitional phases where fracture growth patterns are less distinct. The overlapping regions underscored the constraints of unsupervised approaches and indicated the necessity for additional enhancement using supervised learning models. An extra insight was derived from the analysis of cluster centroids and the examination of representative signals from each group. The centroids of each cluster, when reintroduced into the feature space, enabled us to discern prototype signal patterns for each phase of crack formation. These prototypes serve not just for classification but also for creating interpretable models that highlight the fundamental behavior of materials. The integration of clustering and visualization facilitated the identification of mislabeled or outlier data, enhanced dataset quality, and provided direction for expert evaluation.

## 6. Fatigue Crack Length Estimation Using CNN

**Figure 9:**
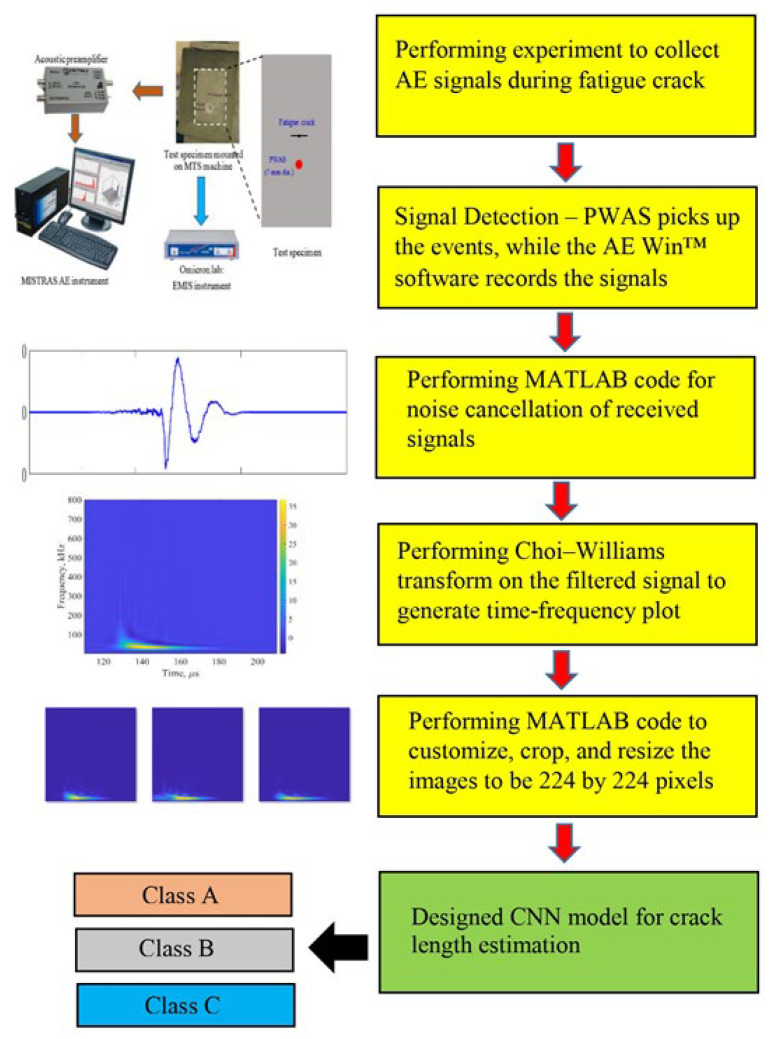
The schematic illustrates the proposed CNN model.

All acoustic emission (AE) data were collected from a single specimen. The dataset was split following a chronological, specimen-wise manner rather than by random shuffling of individual AE hits. Specifically, AE hits were first grouped into three classes (Table 1) based on continuous fatigue cycle intervals corresponding to distinct crack length stages. Each class-specific dataset was then randomly divided into three subsets: 70% for training, 10% for validation, and 20% for testing. Consequently, no temporally adjacent or overlapping AE hits were shared among the three subsets, thereby preventing temporal leakage. As outlined in Section 4, the image dataset corresponding to each crack length was compiled. The images are categorized into three classes: class A (208 images), class B (560 images), and class C (520 images), as shown in Table 1. Figure 9 demonstrates the implementation of the proposed CNN model for estimating crack length through the classification of three distinct classes.

### 6.1. CNN-Based Feature Extraction

At this point, a Convolutional Neural Network (CNN) model is built to automatically get high-level features from the time-frequency representations of AE image-based data. There are many convolutional and pooling layers in the architecture that help the model understand spatial hierarchies in the input images. After that, there are fully connected layers that help the model abstract features. The CNN is made to find local patterns and correlations in the converted AE data. These are very important for telling the difference between various phases of crack propagation. The model learns from labeled data that fits into one of three known fatigue crack length classes (see Table 1). The intermediate activations from certain layers are then used as feature vectors for grouping and classification. To prevent overfitting and improve the model’s robustness and generalization to unseen data, aggressive data augmentation techniques were applied in real-time during the training phase. Given that the time-frequency representations (e.g., spectrograms) are fundamentally images with a physical interpretation, geometric transformations must be applied carefully to generate new AE-image-based samples. The primary augmentation strategies included: random horizontal shifting (time-axis translation), which simulates slight variations in the temporal onset of an AE event; moderate vertical shifting, which accounts for minor shifts in resonant frequencies; and random, small-angle rotations, which introduce invariance to minor alignment discrepancies or baseline tilts in the signal. Crucially, operations that would destroy the physical correspondence of the data, such as vertical flipping or extreme rotations, were avoided. This augmented dataset, significantly larger and more diverse than the original, forces the CNN to learn invariant, essential features of crack growth stages rather than memorizing spurious, sample-specific artifacts.

Furthermore, Dropout and batch normalization are employed within the network architecture to enhance generalization and stabilize the training process. The custom CNN is used to extract features before classifying them with SoftMax layers or as input to clustering techniques, like K-Means, described in Section 5. PCA visualizations are used to test the learned features to see how well they can separate crack stages in a low-dimensional space. The learned features are evaluated using PCA visualizations to assess their ability to separate crack stages in a low-dimensional space.

### 6.2. Custom CNN Architecture

**Figure 10:**
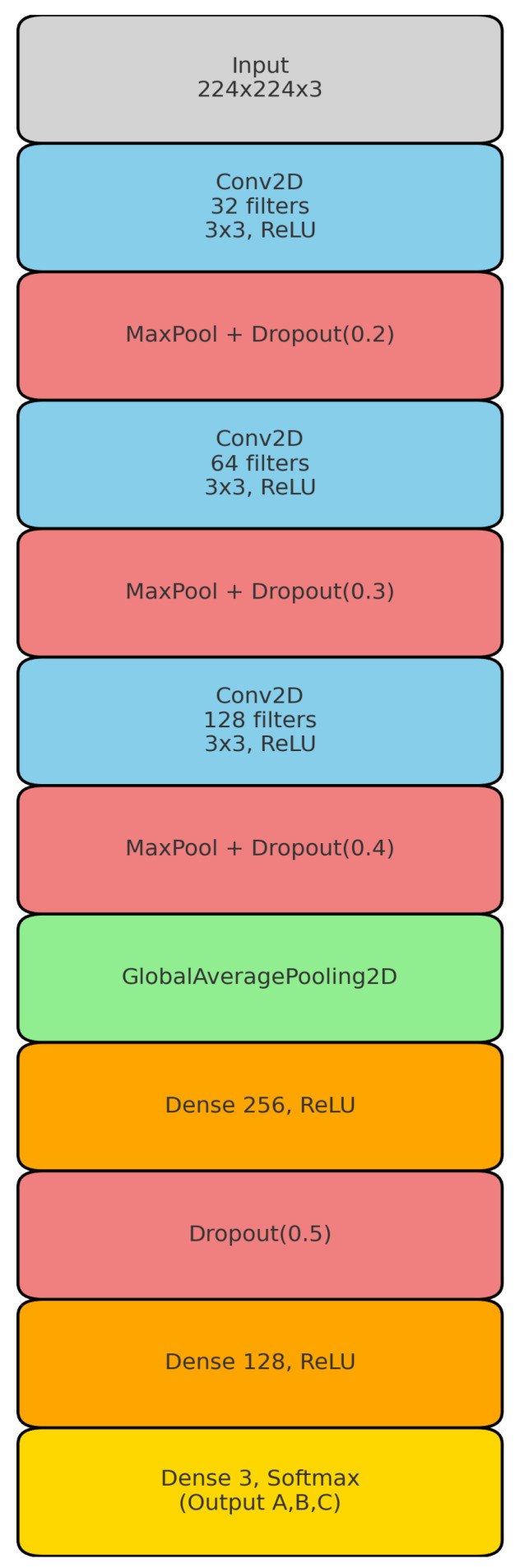
The schematic of the designed CNN-based model.

The convolutional neural network (CNN) model, as shown in Figure 10, has a layered structure. This structure gradually extracts and improves features from input images, which are 224 × 224 × 3 in size. The model’s architecture initiates with several convolutional layers, each employing 3 × 3 kernels and ReLU activation functions; this design facilitates the capture of spatial hierarchy while also preserving non-linearity, hence promoting successful feature learning. The first convolutional layer has 32 filters. This is followed by layers with 64 and 128 filters, respectively, which doubles the depth of the network to help it learn more complicated patterns. Each convolutional block uses the same padding and a stride of 1 to keep the spatial dimensions the same. Then, MaxPooling 2D layers are added to reduce the size of the feature maps, which helps lower the computational cost. To reduce overfitting, dropout layers with rates of 0.2, 0.3, and 0.4 are strategically placed after pooling layers. This approach works by randomly deactivating neurons during the training process.

After extracting features through convolution, the model proceeds to fully linked (dense) layers to do classification. The flattened features then travel through fully connected layers with 256 and 128 units, respectively. Each of these layers uses the ReLU activation function to introduce non-linearity. The last dense layer, which has three units and uses a SoftMax activation function, gives class probabilities for the three target classes: A, B, and C. Including Global Average Pooling 2D before dense layers reduces the number of dimensions while keeping critical spatial information, which improves computing performance. To further regularize the model and prevent neurons from becoming excessively dependent on each other, dropout rates are progressively increased in the following layers, going from 0.2 to 0.5. The introduction of kernel restrictions, even though their specific details are not given, suggests a form of extra regularization. This helps to manage the size of the weights, which in turn promotes better generalization.

This architecture seeks a balance between complexity and simplicity. It uses convolutional layers to find local features and dense layers to make overall decisions. The model’s structure uses common methods for building Convolutional Neural Networks. It uses small 3 × 3 filters to find detailed features, pooling layers to make it less sensitive to where things are in the image, and dropout to help prevent overfitting. The SoftMax function, which is the final layer, produces a probabilistic output. This makes the model ideal for classification tasks with multiple classes. The architecture is generally suitable for picture classification tasks of moderate size. However, its efficiency and accuracy could be improved by using newer techniques like batch normalization or residual connections.

### 6.3. Transfer Learning with Pretrained Models

Transfer learning, which uses pretrained models like VGG16 and ResNet50 [45,46], is used to improve performance and reduce the time needed for training. The models are fine-tuned using the AE dataset. This is done by replacing the final fully connected layers with task-specific layers designed for classifying crack lengths. Initially, the convolutional base layers are kept unchanged. Then, during fine-tuning, they gradually become trainable. This allows for task-specific adjustments while reducing the risk of overfitting. To obtain the best performance, hyperparameters such as the learning rate, batch size, and optimizer type are carefully adjusted. To prevent overfitting and improve stability, early stopping and learning rate schedulers are used. The performance of each pretrained model is compared against the custom CNN, utilizing classification metrics and visualization methods. The results show that transfer learning achieves comparable accuracy while using less training data and requiring less time. This study used the core weights from two pre-trained models: ResNet50V2 and VGG16. The next subsections will describe the architecture and hyperparameters of each model.

#### 6.3.1. ResNet50V2

**Figure 11:**
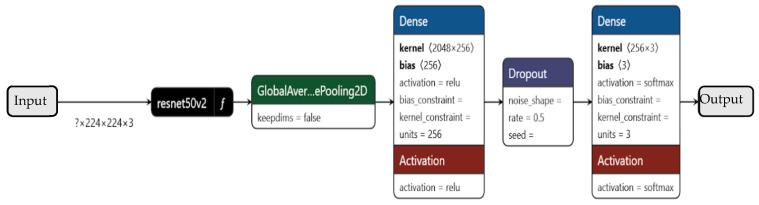
The schematic shows the ResNet50V2 CNN architecture.

ResNet50V2, as shown in Figure 11, is a variant of the Residual Network (ResNet) architecture that introduces identity skip connections to mitigate vanishing gradients in deep networks. Its structure consists of 50 layers, including convolutional blocks with batch normalization and ReLU activation, followed by residual connections that enable smoother gradient flow. Key hyperparameters include the input shape (typically 224 × 224 × 3), batch size (32), and learning rate (1 × 10^−3^) for transfer learning. The model benefits from pretrained ImageNet weights, allowing feature extraction without training from scratch. Fine-tuning involves unfreezing deeper layers while keeping early layers frozen to retain generalized features. Due to its residual design, ResNet50V2 excels in training stability and is well-suited for tasks requiring deep feature extraction.

#### 6.3.2. VGG16

**Figure 12:**
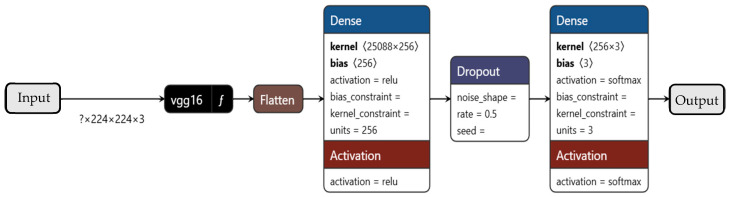
The schematic shows the VGG16 CNN architecture.

VGG16, as shown in Figure 12, is a classic CNN architecture with 16 weight layers, characterized by its simplicity—using only 3 × 3 convolutional filters stacked in blocks, followed by max-pooling and fully connected layers. Its uniform structure makes it easy to implement, but it requires significant computational resources due to its dense parameter count. Key hyperparameters include the input size (fixed at 224 × 224 × 3), batch size (32), and learning rate (1 × 10^−3^ for initial training, reduced to 1 × 10^−5^ for fine-tuning). Unlike ResNet, VGG16 lacks skip connections, making it prone to vanishing gradients in very deep configurations. However, its deep convolutional stacks capture hierarchical features effectively, making it a strong baseline for transfer learning, particularly when computational constraints are not a concern.

## 7. Model Training and Evaluation

Model training is conducted using a stratified training–validation–test split to ensure balanced representation of crack lengths across all subsets. Cross-entropy loss is used for classification, optimized using Adam. To ensure a fair and rigorous comparison between VGG16, ResNet50, and the proposed CNN models, a standardized training protocol was applied to all methods. Each model was trained for up to 100 epochs. To prevent overfitting and determine the optimal stopping point, we implemented a common early stopping criterion that halts the model training process and restores the model’s weights to those from the epoch with the lowest validation loss. This process was monitored using the same fixed validation set for all models. All other hyperparameters (optimizer, initial learning rate, batch size) were individually optimized for each model architecture. The training pipeline includes callbacks to record loss and accuracy metrics, which are later used for plotting learning curves. To evaluate model performance, standard classification metrics are used: accuracy, precision, recall, and F1 score. Confusion matrices are generated to assess the model’s ability to distinguish between different crack length classes. The learning curves show the progression of training and validation accuracy/loss over time, revealing overfitting or underfitting patterns. These visual tools, along with metric values, inform decisions about further tuning or model redesign. The best-performing model is selected based on the highest evaluation scores. Our findings are further analyzed to assess robustness across varied AE signal conditions. This evaluation framework ensures that the proposed method is both accurate and reliable for fatigue crack length estimation.

### 7.1. Hyperparameters and Training Settings

Hyperparameters are the adjustable parameters that govern the training process of a deep learning model, distinct from the model’s internal weights that are learned during training. Key hyperparameters include the learning rate, batch size, number of epochs, optimizer choice, and architecture-specific settings like dropout rates or layer sizes. In this work, the critical hyperparameters are defined as follows: IMG_SIZE = (224, 224), BATCH_SIZE = 32, and EPOCHS = 100, while others are embedded in the model configuration (e.g., Adam optimizer with learning_rate = 0.001). The learning rate is particularly crucial—too high may cause unstable training, while too low may lead to slow convergence. The batch size affects memory usage and gradient stability, and the number of epochs determines how long the model trains, with early stopping implemented to prevent overfitting. Transfer learning-specific hyperparameters include the choice of base model (e.g., VGG16, Resnetv2) and which layers to freeze during initial training.

Training settings in this implementation incorporate several best practices to optimize model performance and efficiency. The work uses data augmentation (rotation, shifts, flips) during training to improve generalization, while validation and test sets use only rescaling. Callbacks like EarlyStopping, ModelCheckpoint, and ReduceLROnPlateau automate critical training decisions: stopping when validation performance plateaus, saving the best model, and dynamically adjusting the learning rate. The training process is split into two phases—initial training with a frozen base model followed by optional fine-tuning of deeper layers—with separate learning rates (1 × 10^−3^ for initial training, 1 × 10^−5^ for fine-tuning) to prevent catastrophic forgetting. The implementation also includes comprehensive evaluation metrics (classification reports, confusion matrices) and systematic logging of results with timestamps, enabling precise performance tracking and comparison across different hyperparameter configurations. These settings collectively balance computational efficiency with model accuracy while maintaining reproducibility.

### 7.2. Visualization of Learning Curves

**Figure 13:**
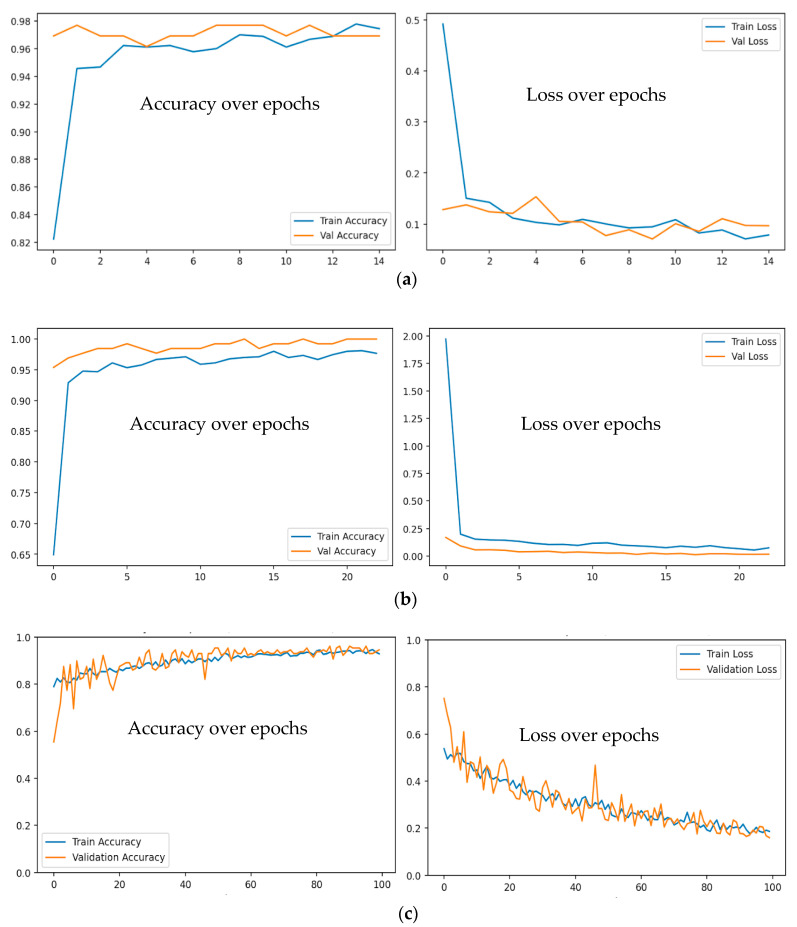
Training progress curves show the accuracy and loss varying with iteration: (**a**) RESNET50v2 CNN; (**b**) VGG16 CNN; (**c**) Proposed CNN Model.

Learning curves are essential tools for diagnosing a model’s training progress and identifying potential issues such as overfitting or underfitting. Figure 13a–c presents two types of key plots: training/validation accuracy and training/validation loss over epochs. The accuracy curve helps assess whether the model is learning effectively, with ideal behavior showing both training and validation accuracy increasing steadily until plateauing. A significant gap between training and validation accuracy suggests overfitting, where the model performs well on training data but poorly on unseen validation data. Conversely, if both curves remain low, the model may be underfitting, indicating insufficient learning capacity or the need for better feature extraction.

The loss curve offers supplementary information by showing how well the model minimizes its prediction error during training. A model that has been trained well should show a decrease in loss for both the training and validation sets, and then it should stabilize. If the validation loss starts increasing while the training loss continues to decrease, it signals overfitting—the model is memorizing the training data rather than generalizing. The work also includes early stopping and learning rate reduction callbacks to automatically halt training if validation performance degrades, ensuring efficient use of computational resources. The model training is halted because of the early stopping flag. The early stopping is presented in Figure 13a,b. By analyzing these curves, practitioners can fine-tune hyperparameters, adjust model complexity, or implement regularization techniques to improve generalization.

### 7.3. Evaluation Metrics

Evaluating a machine learning model’s performance requires robust metrics that measure different aspects of its predictive ability. Accuracy is the most intuitive metric, representing the proportion of correct predictions (both true positives and true negatives) out of all. While useful for balanced datasets, accuracy can be misleading in cases of class imbalance, where a model might achieve high accuracy simply by predicting the majority class. For example, in a dataset where 95% of samples belong to class A, a model that always predicts class A would achieve 95% accuracy despite failing to recognize other classes.

Precision and recall [47,48] provide deeper insights into model performance, particularly for imbalanced datasets. Precision measures the fraction of correctly predicted positive instances among all predicted positives (Equation (3)), indicating how reliable the model is when it predicts a given class. High precision means fewer false positives, which is crucial in applications like spam detection, where incorrectly flagging legitimate emails as spam is undesirable.

(3) $$\mathrm{Precision}=\frac{TP}{TP+FP}$$

Recall, also known as sensitivity, counts the proportion of actual positive cases that the model properly identifies, as shown in Equation (4). This metric is especially essential in medical diagnosis, where failure to find a true positive, such as missing an illness, could have serious effects.

(4) $$\mathrm{Recall}=\frac{TP}{TP+FN}$$

However, precision and recall typically have a trade-off; improving one can lead to a decline in the other. To balance these two measures, the F1-score [47,48] is used. This score is the harmonic mean of precision and recall, as shown in Equation (5). The F1-score is particularly useful when the classes are imbalanced. This is because it penalizes models that perform well on one metric, like high precision but low recall, while neglecting the other. A high F1-score implies that the model performs well, balancing the reduction in both false positives and false negatives.

(5) $$F1\;\mathrm{score}=\frac{2\times Precision\times Recall}{Precision+Recall}$$

In the presented work, these metrics are systematically computed using a classification report, which generates precision, recall, and F1-scores for each class along with macro and weighted averages. Additionally, confusion matrices visually represent true positives, false positives, true negatives, and false negatives, helping identify which classes the model struggles with. Together, these metrics provide a comprehensive evaluation of model performance, ensuring that accuracy alone does not mask potential weaknesses in classification tasks.

### 7.4. Confusion Matrix Analysis

A confusion matrix provides a detailed breakdown of a classification model’s performance by comparing predicted labels against true labels across all classes. The confusion matrices are generated for the training, validation, and test sets, offering a clear visualization of correct classifications (diagonal entries) and misclassifications (off-diagonal entries). By analyzing these matrices, we can identify specific patterns of errors—such as which classes are frequently confused with one another—helping diagnose weaknesses in the model. For instance, if class A is often misclassified as class B, these behaviors may indicate overlapping features between the two classes or insufficient training examples for class A. The use of Seaborn’s heatmap (version 0.13.2) enhances readability, with darker blues highlighting higher counts, making it easy to spot dominant trends in prediction errors.

Beyond simple accuracy metrics, confusion matrices reveal critical insights into class-specific performance. Metrics like precision, recall, and F1-score can be derived directly from the matrix by examining true positives (TP), false positives (FP), and false negatives (FN) for each class. For example, a high count of false negatives in a particular class suggests the model struggles to detect that class, while many false positives indicate overconfidence in incorrect predictions. The systematic generation of confusion matrices for all datasets (train, validation, and test) ensures consistent evaluation, helping verify whether the model generalizes well or suffers from data leakage. By combining this analysis with learning curves and classification reports, we become familiar with the model’s strengths and limitations in real-world scenarios.

## 8. Result and Discussion

### 8.1. Performance Comparison Across Models

**Figure 14:**
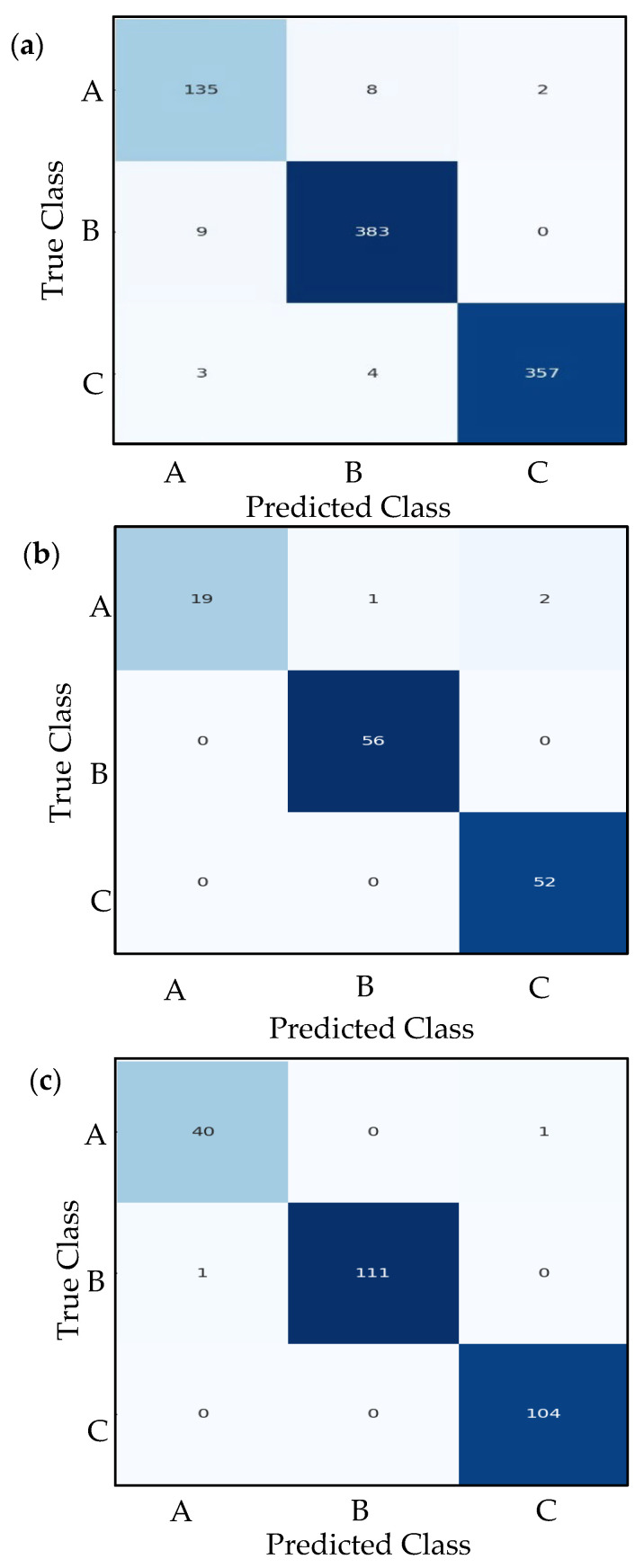
Measured confusion matrices of RESNET50v2 CNN; (**a**) Training confusion table; (**b**) Validation confusion table; (**c**) Testing confusion table.

**Figure 15:**
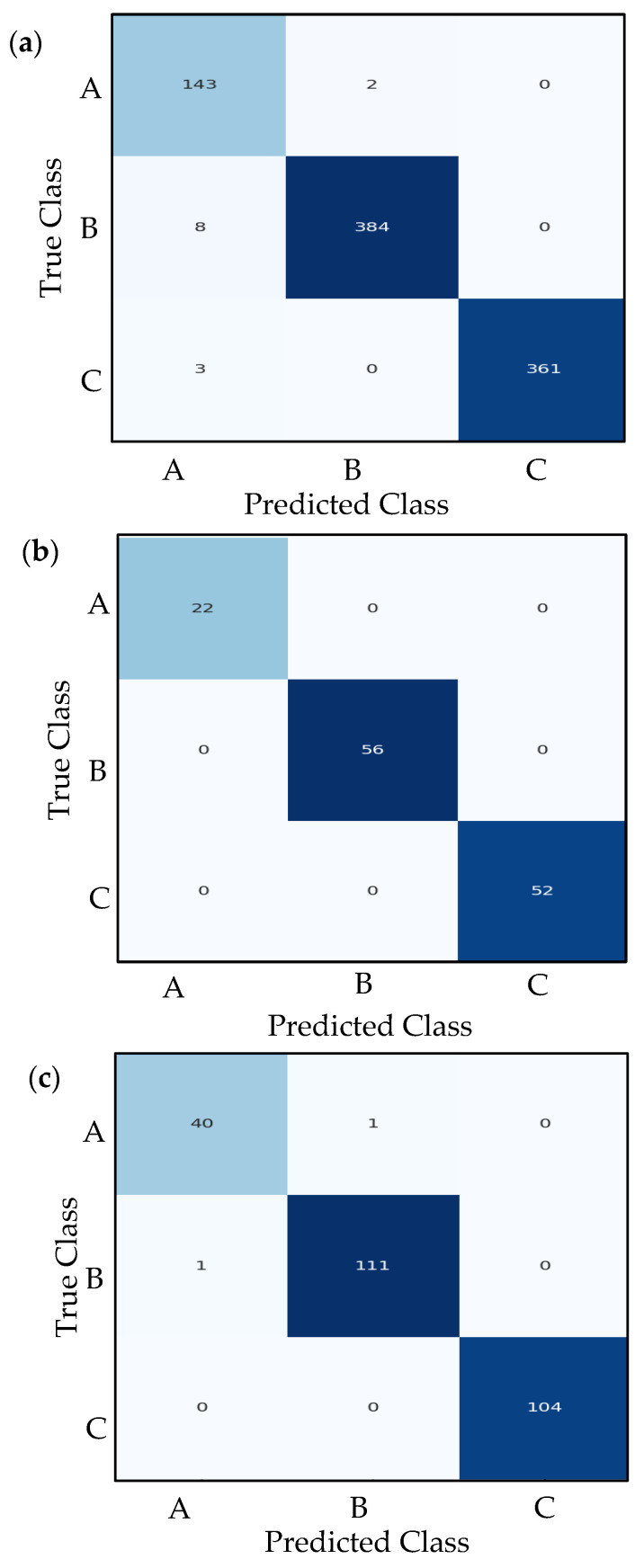
Measured confusion matrices of VGG16; (**a**) Training confusion table; (**b**) Validation confusion table; (**c**) Testing confusion table.

**Figure 16:**
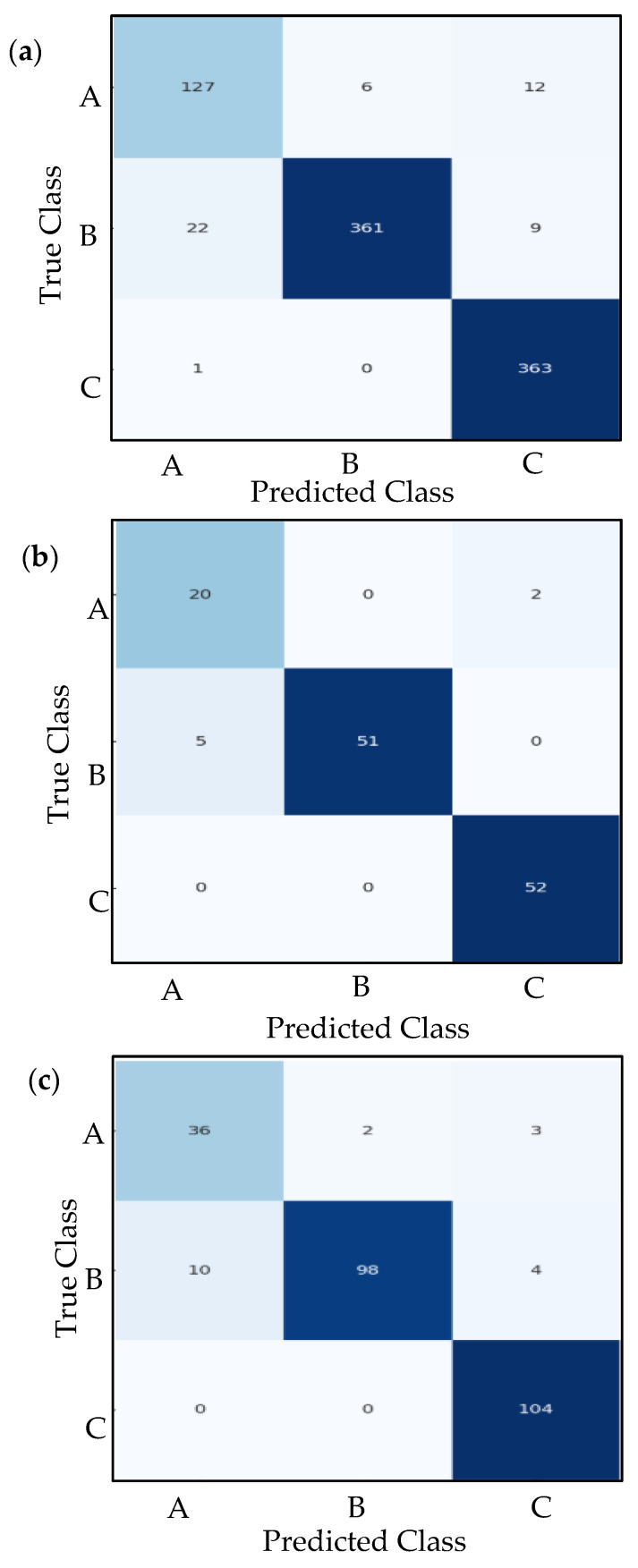
Measured confusion matrices of Proposed CNN; (**a**) Training confusion table; (**b**) Validation confusion table; (**c**) Testing confusion table.

The performance of the three models can be analyzed and compared systematically. The ResNet50v2 (see Figure 14 and Table 2) and VGG16 (see Figure 15 and Table 3) models exhibit comparable overall accuracy, each attaining approximately 99.22% on the test set. This demonstrates that both pre-trained deep architectures are highly effective in differentiating among the three classes (A, B, and C) for this particular task. The performance metrics demonstrate consistency across precision, recall, and F1-score for each class. VGG16 achieves perfect scores for class C, while ResNet50v2 exhibits a near-perfect balance, with a minor trade-off between precision and recall for classes B and C. The proposed CNN-based model demonstrates a lower yet still substantial overall accuracy of 93% (see Figure 16 and Table 4). A comprehensive class-wise analysis indicates the performance disparity. The proposed model for class A exhibits an F1-score of 0.83 (see Table 4), which is significantly lower than the nearly perfect score of 0.98 attained by both ResNet50v2 and VGG16. This low performance could be a result of an imbalanced dataset, where the number of data samples of class A (208) is less than the data samples of classes B and C. This imbalance may influence the observed performance differences across models. The decrease is mainly attributed to a lower precision of 0.78, signifying that the proposed model accurately predicts class A 78% of the time, in contrast to 97.5% for the alternative models. The recall for class A is 0.88, which remains below that of the benchmark models. Performance variations can be ascribed to the architectural benefits of the pre-trained models. ResNet50v2 and VGG16 are robust architectures that have been pre-trained on a large dataset (ImageNet), equipping them with a solid foundational comprehension of visual features. This enables precise optimization for the specific classification task, resulting in significant success. The proposed CNN model, although simpler and trained from scratch, does not possess extensive pre-training or inherent hierarchical feature extraction capabilities. As a result, it encounters greater difficulties, especially regarding the nuances of class A, resulting in an increased number of false positives for that category and an overall less balanced performance. In conclusion, the proposed CNN model attains an accuracy of 93%, yet it is notably surpassed by transfer learning methods employing ResNet50v2 and VGG16. The benchmark models establish a high standard, exhibiting nearly flawless classification metrics. In tasks necessitating high reliability, particularly for class A, pre-trained models demonstrate clear superiority. The findings indicate that utilizing transfer learning with deep pre-trained architectures is a more effective approach for this issue compared to developing and training a conventional CNN from scratch.

### 8.2. Analysis of Classification Reports

This section provides a detailed discussion about the performance of three models: The RESNET50v2, VGG16, and the proposed model.

1- The RESNET50v2 model demonstrates exceptionally high performance, nearly reaching perfection on this test set. The accuracy, macro average, and weighted average F1-scores are all approximately 0.992, indicating a very balanced and well-performing model across all classes. The class-wise performance of the three classes can be illustrated as follows:
  - Classes B and C: Performance is near-flawless. Class C has a perfect recall of 1.0, meaning it did not miss a single sample from this class. Class B has a near-perfect precision of 1.0, meaning every sample it predicted as Class B was correct.
  - Class A: Shows a slight, uniform dip in precision, recall, and F1-score (0.976). This suggests that the model made a couple of errors, specifically with Class A, both in missing a few (lower recall) and misclassifying a few others as Class A (lower precision).
2- The VGG16 model’s performance is virtually identical to RESNET50v2, also performing exceptionally well with a tiny variation. Like RESNET50v2, its overall accuracy and average scores are 0.992. The class-wise performance of the three classes is slightly different, as illustrated below:
  - Class C: Shows perfect precision, recall, and F1-score (1.0).
  - Class A: Identical to RESNET50v2, with scores of 0.976.
  - Class B: The scores (0.991) are slightly different from RESNET50v2’s Class B (which had a perfect precision but slightly lower recall). Here, precision and recall are equal, indicating a different, but equally minor, type of error distribution for this class.

The performance difference between these two pre-trained models is negligible (a difference of <0.004 in any average metric). Both are top performers on this dataset.

3- The proposed CNN model performs very well but is a clear step below the two pre-trained models. The accuracy and weighted average F1-score are at 0.93. This is very good, but significantly lower than the 0.99 achieved by RESNET50 and VGG16. The performance of the proposed model can be summarized as follows:
  - Class C: Excellent performance, with perfect recall (1.0) and a high F1-score (0.97), on par with the top models.
  - Class B: Good performance (F1-score 0.92), but notably lower than the pre-trained models. The recall of 0.88 indicates it failed to identify ~12% of the actual class B samples.
  - Class A: This is the model’s weakest area. An F1-score of 0.83 is significantly lower than the ~0.98 of the other models. The recall of 0.88 is acceptable, but the precision of 0.78 is low, indicating that when it predicts class A, it is incorrect about 22% of the time. This is the primary source of the model’s overall performance gap.

The proposed CNN model is a strong, functional model, but it struggles most with differentiating class A from the others, particularly making false positive errors (predicting A when it is not). Despite targeted augmentation, the suggested CNN’s less complex design may be more prone to acquiring a majority-class bias compared to the fully regularized, pre-trained models. In training, emphasizing accurate categorization of the plentiful, distinct signals from classes B and C results in a more rapid decrease in loss. As a result, the model establishes fewer robust decision limits for class A, rendering it susceptible to misclassifying ambiguous signals from other classes as class A, which further diminishes precision.

## 9. Conclusions and Future Work

### 9.1. Conclusions

We acknowledge that the current research and data processing, which utilizes time-frequency analysis and a CNN interface, were implemented following the collection of experimental data. Therefore, end-to-end runtime evaluation has not been performed in this study. The main conclusion points of this study are as follows:

1. A deep learning–based framework was successfully designed and validated to estimate fatigue crack length from Acoustic Emission (AE) signals.
2. The Choi–Williams transform (CWT) was employed to convert raw AE waveforms into time–frequency representations, which proved to be highly effective input features for Convolutional Neural Networks (CNNs) in capturing complex crack-related signal patterns.
3. Transfer learning models (ResNet50V2 and VGG16) significantly outperformed the custom CNN, achieving approximately 99% classification accuracy, compared to 93% accuracy for the model trained from scratch.
4. The results demonstrate that leveraging pre-trained features from large-scale image datasets is a highly effective strategy for AE-based fatigue crack length estimation.
5. The trained models exhibited excellent precision, recall, and F1-scores across three distinct crack length categories, confirming the reliability of the proposed method in identifying fatigue crack propagation stages.
6. The proposed framework provides a robust, data-driven solution for Structural Health Monitoring (SHM), offering a practical and accurate alternative to traditional non-destructive evaluation techniques with the capability for near real-time crack length assessment.
7. Overall, this work confirms the strong potential of CNN-based analysis of AE signals for automating fatigue fracture monitoring and enabling predictive maintenance strategies in metallic structures.

### 9.2. Future Work

The proposed approach for real-time crack detection will be part of future work that includes a variety of crack geometries, metallic alloys, and loading scenarios.

## Figures and Tables

**Table 1:** The fatigue crack classes are classified based on the crack length. The dataset for each class are presented.

Initial Crack Length (mm)	Final Crack Length (mm)	Class Symbol	Total Samples (Hits)
3.5	5.5	A	208
5.5	7.5	B	560
7.5	9.5	C	520

**Table 2:** The Classification report of the RESNET50v2 test set.

	Precision	Recall	F1-Score	Support
A	0.97	0.97	0.97	41
B	1	0.99	0.99	112
C	0.99	1	0.99	104
accuracy	0.99	0.99	0.99	257
macro avg	0.98	0.98	0.98	257
weighted avg	0.99	0.99	0.99	257

**Table 3:** The Classification report of the VGG16 test set.

	Precision	Recall	F1-Score	Support
A	0.97	0.97	0.97	41
B	0.99	0.99	0.99	112
C	1	1	1	104
accuracy	0.99	0.99	0.99	257
macro avg	0.98	0.98	0.98	257
weighted avg	0.99	0.99	0.99	257

**Table 4:** The Classification report of the Proposed CNN test set.

	Precision	Recall	F1-Score	Support
A	0.78	0.88	0.83	41
B	0.98	0.88	0.92	112
C	0.94	1	0.97	104
accuracy	0.93	0.93	0.93	257
macro	0.9	0.92	0.91	257
weighted	0.93	0.93	0.93	257

## Data Availability

Data is contained within the article.
